# Multicenter randomized controlled trial on the comparison of multi-family therapy (MFT) and systemic single-family therapy (SFT) in young patients with anorexia nervosa: study protocol of the THERAFAMBEST study

**DOI:** 10.1186/s13063-019-3347-y

**Published:** 2019-04-30

**Authors:** Benjamin Carrot, Jeanne Duclos, Caroline Barry, Leslie Radon, Anne-Solène Maria, Irène Kaganski, Zorica Jeremic, Vesper Barton-Clegg, Maurice Corcos, Malaïka Lasfar, Priscille Gerardin, Aurélie Harf, Marie-Rose Moro, Corinne Blanchet, Nathalie Godart

**Affiliations:** 1 Psychiatry Unit, Institut Mutualiste Montsouris, Paris, France; 2 CESP, INSERM, UMR 1018, University Paris-Sud, UVSQ, University Paris-Saclay, Villejuif, France; 3 Univ. Lille, CNRS, CHU Lille, UMR 9193 - SCALab - Cognitive and Affective Sciences, F-59000 Lille, France; 4 Department of Addiction, Eating Disorders Unit, Paul Brousse Hospital, Villejuif, France; 5 Department of Clinical Psychology, Psychopathology, Psychoanalysis - EA 4056 (PCPP), University of Paris Descartes, Paris, France; 6 Department of Medical Pediatrics and Child and Adolescent Psychiatry, Rouen University Hospital and Rouvray Hospital, University of Rouen, Rouen, France; 7 Maison de Solenn, Maison des Adolescents, Hôpital Cochin, Paris, France; 8 UFR des Sciences de la Santé Simone Veil (UVSQ), Versailles, France; 9 Fondation de Santé des Etudiants de France, Paris, France

**Keywords:** Anorexia nervosa, Adolescents, Systemic family therapy, Multi-family therapy, Randomized controlled trial, Cost-efficiency analysis

## Abstract

**Background:**

Anorexia nervosa (AN) is a serious psychiatric illness that begins most of the time during adolescence. An early and efficacious intervention is crucial to minimize the risk of the illness becoming chronic and to limit the occurrence of comorbidities. There is a global consensus on optimal treatment for adolescents suffering from AN: international guidelines recommend single-family therapy that involves the patient and his/her family. Several family therapy approaches have been developed to date. However, these approaches, which imply a direct questioning of intrafamilial dynamics, are not suitable for all patients and families, and the rates of dropout or poor response to treatment remain quite high. A modality of family therapy has been adapted to AN, known as multi-family therapy (MFT), which consists in bringing together several families whose children suffers from the same illness. Objectives of the present randomized clinical trial are to evaluate whether the implementation of MFT in a multi-disciplinary treatment program for adolescents with AN is at least as efficacious as the use of systemic single-family therapy (SFT), with respect to the evolution of body mass index and other clinical outcomes 12 and 18 months after the start of treatment. A cost-efficiency analysis will also be conducted.

**Methods:**

One hundred fifty patients meeting the inclusion criteria will be randomly assigned to one of the two treatment groups. Patients and their families will receive 10 sessions of therapy spread over 12 months. Body weight, eating disorder and other psychopathology-related symptoms, quality of family relationships, and family satisfaction with treatment will be evaluated during the treatment and at an 18 months follow-up. A cost-efficiency analysis will also be carried out.

**Discussion:**

We hypothesize that MFT is at least as efficacious as SFT, but at a lesser cost. The identification of possible preferential indications for each technique could help the improvement of therapeutic indications for adolescents suffering from AN and contribute to the earliness of intervention, which is associated with a better outcome.

**Trial registration:**

ClinicalTrials.gov, NCT03350594. Registered on 22 November 2017. IDRCB number 2016-A00818-43.

**Electronic supplementary material:**

The online version of this article (10.1186/s13063-019-3347-y) contains supplementary material, which is available to authorized users.

## Background and rationale

Anorexia nervosa (AN) is a psychiatric disorder included among eating disorders with a reported prevalence in women ranging from 0.5 to 2.2% [1]. Pauci-symptomatic forms of AN (i.e., those in which all the diagnostic criteria for AN are not met or only partially met) are 2 to 10 times more frequent than complete forms of AN and account for up to 60% of the patients treated in specialized centers. AN is often associated with psychiatric comorbidities, such as depression and anxiety [2–4], personality disorders [5] and substance abuse [6]. The cost of eating disorders is considerable, as much from individual, familial, and social viewpoints as from an economic viewpoint [7, 8].

According to main international guidelines, involvement of the family in the treatment of adolescents, by means of family therapy, is the recommended therapeutic option [9–11]. Several familial approaches have been developed and implemented over the last decades, among them the Maudsley family-based treatment [12], which is the most commonly implemented form of family treatment to date, and systemic single-family therapy (SFT), which we use in our health care institutions [13]. These modalities have been widely evaluated (see [14] for a review). However, even if studies show better efficacy than other treatments, a significant number of patients do not respond well to this treatment. Across all studies, remission rates are still under 50% 12 to 18 months after the start of treatment. Some families (patients and/or parents) seem reluctant to accept a single-family therapy project, probably because they fear that their family functioning will be challenged or because of an excessive closeness in the therapeutic or family relationships. And even if it is accepted, the single-family therapy approach can fail, particularly as a result of strong resistance to change, substantial family dysfunctions, or serious individual pathologies.

Moreover, economic considerations motivate clinicians and researchers to develop new treatments or improve existing ones to improve efficacy and cost-effectiveness, notably in helping prevent hospitalization, which dramatically increases costs of treatment.

Multi-family therapy (MFT) was thus adapted for adolescents with AN and their families. MFT consists in bringing several families together (generally four to seven) faced with the same pathology (psychiatric or physical) in order to create a therapeutic framework and a social network, or “care community.” This idea came from Laqueur, who believed that the presence of other families could help with the issue of independence, a source of conflict, via an identification process with other families [15, 16]. MFT has been applied to various psychiatric pathologies, such as schizophrenia [17, 18], depression and bipolar disorder [19, 20], and post-traumatic stress disorder [21]. In AN, the first experiences of MFT were published in the late 1980s in Denmark and the USA [22, 23]. The approach was formalized and manualized several years later by the Maudsley team as an intensive outpatient treatment [24–27]. Since that time, several teams have implemented MFT in their clinical settings as an alternative to hospitalization and/or single-family therapy [28–35]. If results from observational studies show that intensive MFT approaches present valuable elements regarding efficacy [27, 28, 33–36] implementation of such a treatment is not always possible in terms of availability and motivation of families or financial and human resources. This is why, in France, other teams have adapted and integrated MFT into their pre-existing standard treatment for children and adolescents suffering from AN [37–40]. There are encouraging results from a first observational study showing good efficacy and high satisfaction with treatment [38]. Implementing MFT could thus increase the therapeutic resources available to the patients and their families.

Even if the international literature upholds MFT as a non-negligible therapeutic tool to prevent relapse and as an alternative to hospitalization, to date and to our knowledge, no study has evaluated MFT in a less intensive format than the Maudsley approach (i.e., outpatient setting) in comparison to another form of family treatment. The THERAFAMBEST study aims to provide new evidence regarding MFT in adolescents with AN and to compare this approach to SFT as it is classically proposed in our care setting, in terms of efficacy, cost, family satisfaction, and clinical outcome.

## Objectives

### Primary objective

The main objective of this trial is to examine whether the implementation of MFT in a multi-disciplinary treatment program for adolescents with AN is at least as efficacious in terms of body mass index (BMI) evolution as the implementation of SFT, 12 months after the start of treatment.

### Secondary objectives

Secondary objectives are as follows:

1. To examine whether adding an MFT to a multi-disciplinary treatment program for adolescents with AN is at least as efficacious as SFT, 12 months after the start of treatment, in terms of clinical evolution, evaluated by other clinical outcomes (global clinical state, weight status, menstruation, eating disorder symptoms, anxiety, depressive and obsessive symptoms, social adaptation, family relationships, number and duration of hospitalizations after inclusion, subject and parents’ satisfaction, and parental relief)
2. To study the cost of implementation of each technique in a multi-disciplinary treatment program for adolescent AN, through a cost-efficiency analysis
3. To identify possible preferential indications for each technique with the help of moderating and prognostic factors for the evolution of AN in psychiatric, physical, and social terms
4. In case of the non-inferiority of MFT, to test its superiority in comparison with SFT
5. To carry out a follow-up 6 months after the end of treatment on the same evaluation criteria.

## Hypotheses

We hypothesize that MFT is at least as efficacious as SFT in terms of weight and clinical evolution, and will be better accepted and not as costly in our treatment program.

We also hypothesize that some patient profiles will have better indications for one or another form of therapy.

## Methods

### General design aspects

**Fig. 1:**
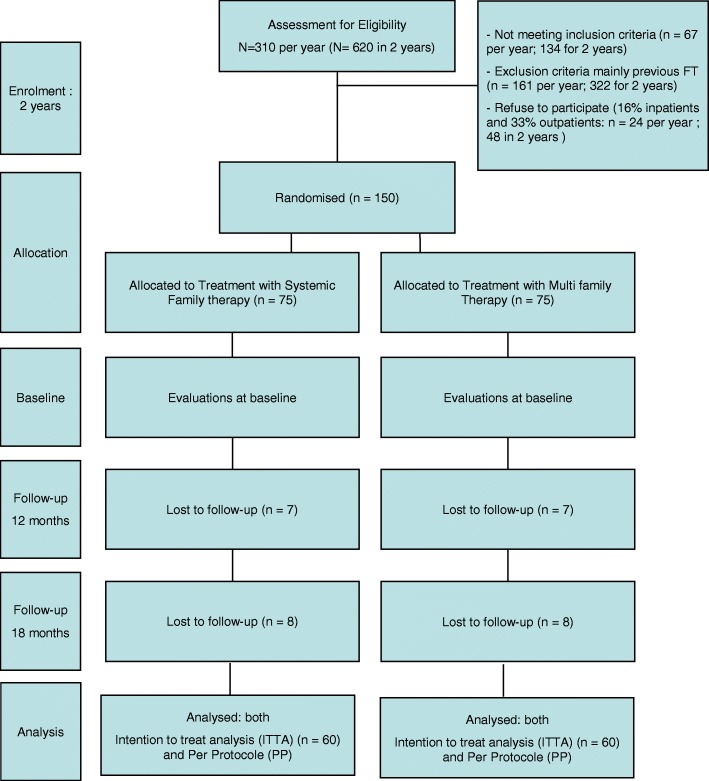
THERAFAMBEST provisional flowchart

This study is a non-inferiority, multi-center, prospective, parallel-group, randomized clinical trial, comparing SFT and MFT with respect to the efficacy in improving patients’ BMI after 12 months of treatment and at 18 months follow-up. The provisional flowchart is presented in Fig. 1. The Standard Protocol Items: Recommendations for Interventional Trials (SPIRIT) checklist is provided in Additional file 1.

The total study duration will be 4 years. The participation duration for study subjects will be 18 months, i.e., a 1-year treatment period followed by an evaluation 6 months after the end of treatment.

### Cost-efficiency analysis

An economic assessment is planned in order to take into account, in comparing the two interventions under study, the cost of the interventions, difficulties in implementing them, the various treatments and forms of support that families of patients with eating disorders resort to, and the informal assistance provided by the patients’ close circle.

A cost-efficiency analysis will be conducted on the costs generated by the interventions with a perspective on their impact on the patients’ health and the families’ quality of life. This methodological choice results from the multi-dimensional consequences of the illness on the patients’ physical and psychiatric health and social integration, and on their families. These consequences cannot be apprehended by a single indicator.

### Participants

#### Inclusion criteria

All patients treated in outpatient care or hospitalized for AN in one of the three study sites (Pr. Corcos’ unit at Institut Mutualiste Montsouris, Paris; Pr. Moro’s unit at the Maison des Adolescents, Cochin Hospital, Paris; Pr. P. Gerardin’s unit in Rouen University Hospital) will be offered participation in the study if they (1) have a Diagnostic and Statistical Manual of Mental Disorders fifth edition (DSM-5) diagnosis of AN or a diagnosis of Eating Disorder Not Otherwise Specified (EDNOS), (2) are aged between 13 to 19 years, (3) have been diagnosed with AN or EDNOS before 19 years old, (4) benefit from a social welfare program, (5) live, and their family lives, in Paris and its suburbs or Rouen or in the vicinity of Rouen.

#### Non-inclusion criteria

Patients with one of the following diagnoses will not be included in the study: (1) psychotic state, (2) mental deficiency, (3) organic brain disorder, (4) metabolic pathology interfering with eating or its regulation (diabetes being the most common one). Patients who are not fluent in French will not be included in the study. Patients who have undergone family therapy before the current treatment will not be included in the study.

Non-included subjects will be listed along with the causes of their non-inclusion.

#### Randomization

All patients who give consent for participation and who fulfill the inclusion criteria will be randomized. Randomization will be centralized and stratified according to the status of treatment at inclusion (outpatient care or hospitalization) and the center. A randomization list will be generated by computer, with blocks of variable sizes, by a statistician independent from the group recruiting or taking care of the patients. The allocation ratio will be 1:1. Results of the randomization will be accessible on the comprehensive software used for the trial (CleanWeb™). Family therapy (MFT or SFT) will start during the month following the randomization.

#### Blinding

Only therapists will receive information about group allocation, as well as patients and families. Assessors will be blinded to the treatment group and will use standardized research questionnaires. Data analysts will also be blinded to the treatment group. Therapists will not be involved in the assessment of treatment outcomes.

### Interventions

After their inclusion in the trial, patients and their families will receive either SFT or MFT. For inpatients, intervention will be proposed after the first half of hospitalization (midway between weight at admission and target weight for discharge). For outpatients, intervention will be proposed when the patient’s clinical situation is stable with no indication for a hospitalization. This methodological choice was based on the fact that the introduction of family therapy before nutrition rehabilitation is not beneficial and could even impede the psychotherapeutic process [41]. Treatment as usual will not be otherwise modified.

#### Treatment as usual

Patient follow-up will be carried out as is usually the case in outpatient care or hospitalization, in compliance with the Health Authorities’ recommendations [10] on indications for the level of treatment to be implemented. Multi-disciplinary care is proposed, involving nurses, psychologists, psychiatrists, general practitioners, gynecologists, rheumatologists, dieticians, occupational therapists, and social workers. Targets for treatment include eating disorder symptomatology, consequences of starvation, psychological disorders, and family interactions. A coordinating psychiatrist coordinates the treatment. For a full description of inpatient and outpatient treatment modalities in our structures, see [42–44].

#### Common characteristics of interventions

SFT as well as MFT sessions will be conducted by dyads of specifically trained psychologists and/or psychiatrists on the basis of a treatment manual. Rhythm of therapy will be 10 sessions, spread over 12 months (approximately 1 session per month).

#### Systemic single-family therapy (SFT)

SFT was developed by our team as a component of a multi-dimensional care program (for a detailed description, see [13, 45, 46]). The therapy involves the patient, the parents, and siblings over the age of 6 living at home. Sessions will last approximately 1 h and 30 min.

Main objectives of SFT include the following:

- To develop and maintain a therapeutic alliance,
- To redefine areas of individual responsibility and clarify generational boundaries, to strengthen parents' capacity to exercise their parental authority,
- To rebuild the family’s abilities to protect and support,
- To enable appropriate expression and management of conflict and rivalry,
- To allow the family to rediscover its own resources and strengths, needed to adapt to the changes associated with adolescence,
- To rebuild a collective sense of family identity, which is not based on the sacrifice of family members’ personal needs,
- To develop the patient’s autonomy.

To achieve these objectives, therapy is carried out following two main axes, diachronic and synchronic, i.e., regarding the past, the present, and the future. Sessions focus on the family's dynamics as a whole. Eating behaviors are not addressed directly by the family therapists, but by the referring psychiatrist. Without denying the importance of the personal suffering, nor the somatic and biologic aspects of the pathology, emphasis is placed on interactions which take place around the symptoms.

#### Multi-family therapy (MFT)

Each session will involve 5 to 7 families of patients suffering from AN including the parents and non-systematically the siblings. Sessions will last 3 h.

 MFT follows the integrative model practiced by Cook-Darzens, which includes elements from the Maudsley approach [37]. MFT will be conducted according to three main dimensions: psychoeducative dimension, support group dimension and family therapy dimension. Four main themes are addressed: (1) understanding and managing AN, (2) family relations and family identity, (3) overcoming social isolation, (4) values, beliefs, and perceptions.

In addition to the general objectives of SFT, objectives of MFT include the following:

- To improve communication and interaction between the different family members,
- To overcome the sense of isolation and the feeling of shame, by creating a sense of solidarity between families facing the same illness,
- To foster mutual learning and support, by sharing experiences,
- To develop competencies to fight against the pathology,
- To develop parental empathy toward the patient, to decrease attitudes of hostility and rejection,
- To maintain an attitude of hope and realistic optimism,
- To avoid stagnation in relationships between carers and patients.

While some of the objectives are achieved naturally by the structure and dynamics of the group, other objectives are encouraged by specific interventions and therapeutic strategies and techniques. MFT uses a wide range of techniques and approaches, reflecting the conceptual diversity that underlies it. MFT tools are based on cognitive behavioral (psychoeducational approach), systemic (group approach and family therapy), psychodynamics (differentiation process through identification with other families, learning by analogy) and medical family therapy concepts and practices (adaptation to processes of illness). As described by some authors [28], this mix of practices creates a “greenhouse effect” that promotes openness and catalyzes change. MFT is based on the hypothesis that the intensity of the program creates opportunities for each family to witness extreme emotions and receptiveness [24, 28]. This process is an important factor in the evolution of family dynamics, allowing families to change some of the behaviors that had so far restricted the scope for change and growth.

### Training and supervision

Aside from training in the use of each technique, therapists will follow a training program on the contents of the therapeutic manuals used in the trial. All therapists will be supervised with video feedback. An assessment of the conformity of the therapy techniques for each therapist dyad will be carried out randomly during therapy for each family via an assessment coding sheet based on recordings from four sessions, each from one block (1–3, 4–9, 10–12 months).

### Instruments

**Fig. 2:**
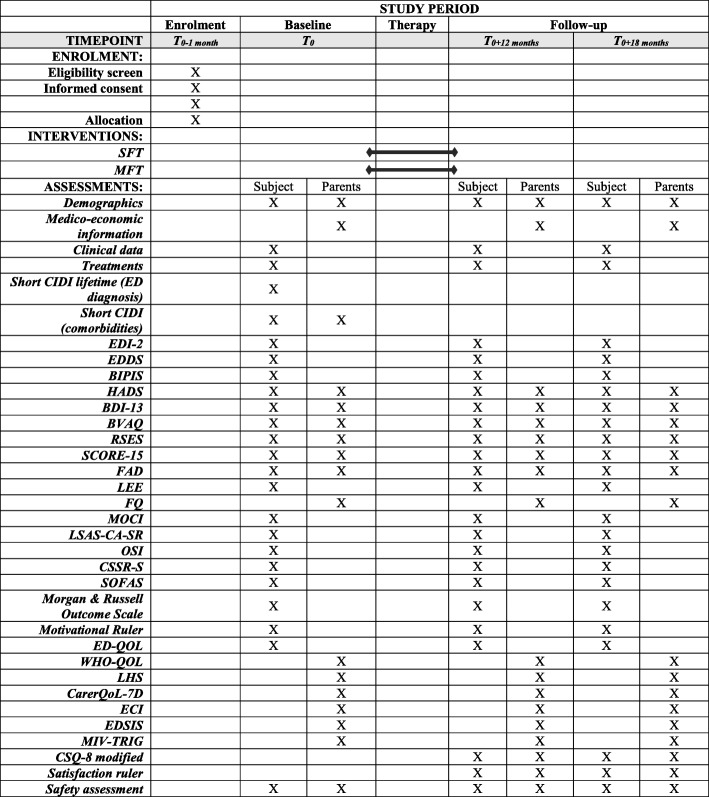
Schedule of enrollment, interventions, and assessments (SPIRIT figure)

A summary of the evaluations proposed to patients and their parents during the study (at inclusion, after 12 months, and after 18 months) is presented in Fig. 2. Evaluations are detailed in the following paragraphs.

Apart from the diagnostic instrument Composite International Diagnostic Interval, short form (Short-CIDI), which will only be administered only at baseline for diagnostic purposes, and the Client Satisfaction Questionnaire (CSQ-8), which will not be administered at baseline, all instruments will be administered at baseline and repeated at 12 and 18 months post randomization.

#### Clinical and demographic information

The patient’s demographic information will be collected through a self-report questionnaire, and clinical information (e.g., history of the illness, previous and current treatments) will be collected through a semi-structured interview. Weight will be measured with the subject wearing only underwear on the same calibrated scales. Stature will be measured using a stadiometer.

Parents’ demographics and medical-economic information will be collected through a self-report questionnaire complemented by a semi-structured interview.

#### Eating disorder and diagnosis of comorbidities

The relevant sections of the Short-CIDI [47] will be used for the diagnosis of eating disorder and comorbidities, including major depression, anxiety disorders (generalized anxiety disorder, post-traumatic stress disorder, social phobia, panic disorder), and substance use disorders.

#### Eating disorder symptomatology

The Morgan-Russell Global Assessment Outcome Schedule (GOAS, [48–50]) evaluates the clinical state over the past 6 months using five clinician-rated subscales assessing Nutrition, Menstruation, Mental state, Psychosexual functioning, and Socioeconomic state. For each item, scores range from 0 to 12. A score for each subscale and an average outcome score can be calculated. Higher scores indicate better functioning. A global outcome category can also be defined [48]. A good outcome is characterized by a weight greater than 10th percentile and the presence of menstruation. An intermediate outcome is characterized by a weight greater than 10th percentile but amenorrhea (absence of menstruation for at least the past 3 months). Finally, a poor outcome is characterized by a weight bellow the 10th percentile and/or the presence of bulimic symptoms.

The Eating Disorder Inventory 2 (EDI-2, [51]) is a 91-item self-report questionnaire used to assess the presence of eating disorders (AN restrictive or binge purging types, bulimia nervosa, EDNOS, binge eating disorder). Each item is rated on a Likert-type scale, from “always” to “never.” EDI-2 comprises 11 subscales: Drive for Thinness, Bulimia, Body Dissatisfaction, Ineffectiveness, Perfectionism, Interpersonal Distrust, Interoceptive Awareness, Maturity Fears, Ascetism, Impulse Regulation, and Social Insecurity. Higher scores indicate higher levels of symptoms.

The Eating Disorder Diagnostic Scale (EDDS, [52]) is a 22-item self-report questionnaire used to assess the presence of anorexia nervosa, bulimia nervosa, and binge eating disorder. The scale consists of a combination of Likert scores, yes/no scores, frequency scores, and open-ended questions. The EDDS consists of a diagnostic scale and a continuous eating disorder symptom composite scale.

The Body Image Psychological Inflexibility Scale (BIPIS, [53]) is a 16-item self-report questionnaire that evaluates psychological inflexibility as it relates to body image distress. Each item is rated on a Likert scale, ranging from 1 (“never true”) to 7 (“always true”). Higher scores indicate higher levels of psychological inflexibility in body image.

#### Anxious and depressive symptomatology

The Hospital Anxiety and Depression Scale (HADS, [54, 55]) is a self-report questionnaire comprising 14 items, among which 7 evaluate depression level and the remaining ones evaluate anxiety level. Higher scores indicate a higher level of symptoms for each dimension.

The Beck Depression Inventory, 13 items (BDI-13, [56, 57]) is a self-report questionnaire measuring the level of depression over the past 7 days. Higher scores indicate a higher level of depressive symptoms.

The Liebowitz Social Anxiety Scale for Children and Adolescents, self-report version (LSAS-CA-SR, [58, 59]) is a 24-item self-report questionnaire assessing social phobia. Twelve items concern social interaction situations, the other 12 items concern performance situations. Each item is rated for fear level and avoidance level on two Likert scales, from 0 (“none”) to 3 (“severe”) and 0 (“never”) to 3 (“usually”), respectively. In addition to a total score, six subscores can be calculated: Anxiety related to Social Interactions, Performance Anxiety, Total Anxiety, Avoidance of Social Interactions, Avoidance of Performance Situations, and Total Avoidance.

#### Obsessive-compulsive symptomatology

The Maudsley Obsessional Compulsive Inventory (MOCI, [60, 61]) is a 30-item self-report inventory with a yes/no response format. Items refer to the most common complaints of subjects who have obsessional traits. In addition to a total score, four partial scores can be calculated: Checking, Washing, Doubting, and Slowness.

#### Alexithymia

The Bermond-Vorst Alexithymia Questionnaire (BVAQ-B, [62–64]) is a self-report evaluation measuring the level of alexithymia. The questionnaire comprises 20 items assessed on a 5-point Likert scale, from 1 (“totally disagree”) to 5 (“totally agree”), spread across five dimensions: Emotionalizing, Fantasizing about virtual matters, Identifying the nature of one’s emotions, Analyzing one’s own emotional states, and Verbalizing one’s own emotional states. Higher subscores are indicative of a higher proneness to alexithymia.

#### Self-esteem

The Rosenberg Self-Esteem Scale (RSES [65, 66]) is a self-reported measure of general self-esteem, widely used in the general population and in clinical populations. The scale comprises 10 items, of which 5 are reversed, assessed on a 4-point Likert scale (from 1 “strongly agree” to 4 “strongly disagree”). The higher the score, the better the self-esteem.

#### Family functioning

The SCORE-15 Index of Family Functioning and Change is a self-report measure based on the original 40-item version of SCORE [67] which evaluates the quality of family life and is also sensitive to therapeutic changes in family functioning. The SCORE-15 comprises 15 items rated on a Likert scale. The perceptions from each family member over the age of 11 years are recorded. In addition to a total score of family functioning, three subscores can be calculated: Strengths and adaptability, Overwhelmed by difficulties, and Disrupted communication.

The Level of Expressed Emotion (LEE) scale [68] is a 60-item self-report questionnaire with a true/false response format that evaluates the emotional tone of a patient’s most important relationships. Four dimensions are assessed: intrusiveness, emotional response to the patient’s illness, negative attitude toward the patient’s illness, and low level of tolerance and expectations. Besides scores on the four dimensions, a total expressed emotion (EE) score can also be generated, ranging from 60 to 120. Higher scores indicate higher EE.

The Family Questionnaire (FQ, [69]) is a 20-item self-report questionnaire evaluating the EE status, focusing on two dimensions: Critical Comments (CC) expressed by the parent toward his child, and Emotional Overinvolvement (EOI), characterized by intrusive, overprotective, self-sacrificing behavior or exaggerated emotional response to the patient’s illness. The FQ comprises 10 items for each subscale, rated on a Likert scale from 1 (“never/very rarely”) to 4 (“very often”).

The Family Assessment Device (FAD, [70]) is a 60-item self-report questionnaire, based on the McMaster Model of Family Functioning and developed to measure structural, organizational, and transactional characteristics of families. Seven dimensions are evaluated: Affective Involvement, Affective Responsiveness, Behavioral Control, Communication, Problem Solving, Roles, and General Family Functioning. All family members are invited to rate how well each statement describes their family. Higher scores indicate worse levels of family functioning.

#### Quality of life and degree of impairment

The Eating Disorders Quality of Life (EDQOL) scale [71] is a 25-item self-report questionnaire evaluating quality of life in patients with ED symptoms. All items are evaluated on a 5-point Likert scale. Four dimensions of quality of life are assessed: Psychological, Physical/Cognitive, Work/School, and Financial. A total score is also provided. Higher scores indicate a lower quality of life.

The World Health Organization Quality of Life-Bref (WHOQOL-Bref) scale [72, 73] is an abbreviated form of the original WHOQOL-100, which is a self-report questionnaire evaluating the subjective quality of life. The WHOQOL-Bref contains 26 items. Four domains are investigated: Physical Health, Psychological Health, Social Relationships, and Environment.

The London Handicap Scale (LHS, [74]) is a measure of handicap based on its six dimensions as defined by the World Health Organization: Mobility, Occupation, Physical Independence, Social Integration, Orientation, and Economic Self-Sufficiency. The six questions are rated on a 6-point scale. For each item, possible answers are associated with a part utility linked to the level of disadvantage. The sum of all 6 utility values and a constant generates a total score ranging from 0 to 1, 1 representing no handicap and 0 representing maximum handicap.

The Carer Quality of Life, 7 Dimensions (CarerQOL-7D, [75]) is a self-report questionnaire comprising seven statements regarding subjective burden. Five dimensions are negative (relational problems, mental health problems, problems combining daily activities with care, financial problems, physical health problems) and two are positive (fulfillment from caregiving and support with lending care). Participants are asked to indicate whether an item applies to them by filling the blank with one of three possible responses: “no”, “some”, or “a lot”. Reponses are rated 2, 1, and 0, respectively, for negative dimensions and 0, 1, and 2 for positive dimensions. The higher the total score, the better the care situation. Low scores indicate lack of support or fulfillment.

The Social and Occupational Functioning Assessment Scale (SOFAS, [76]) is a clinician-rated scale used to assess social and occupational functioning based on medical conditions. The scale is based on a continuum of functioning, from 0 to 100, with higher scores indicating better functioning.

The Eating Disorders Symptom Impact Scale (EDSIS, [77]) is a 24-item self-report scale developed to assess the impact of eating disorder symptoms and behavior of an ill relative on the other family members. Four dimensions can be evaluated: Nutrition, Guilt, Dysregulated Behavior, and Social Isolation. Each item is rated on a 5-point Likert-type scale from 0 (“never”) to 5 (“nearly always”). Higher scores indicate higher impact of the symptoms on the family.

The Experience of Caregiving Inventory (ECI, [78, 79]) is a 66-item self-report questionnaire evaluating 10 dimensions of the experience of caring for an individual with a severe mental illness. The scale is composed of eight negative subscales*,* i.e., Difficult Behaviors, Negative Symptoms, Stigma, Problems with Services, Effects on the Family, Loss, Dependency, and Need for Backup, and two positive subscales, i.e., Positive Personal Outcomes and Good Aspects of the Relationship with the Patient. An overall score can also be produced. All items are rated on a Likert scale from 0 (“never”) to 4 (“nearly always”). Higher scores indicate greater difficulties in the experience of caregiving.

The Mental Illness Version of the Texas Inventory of Grief (MIV-TIG, [80]) was developed based on the Texas Inventory of Grief [81] for assessing grief in family members of a mentally ill individual. This self-report questionnaire comprises 24 items, 8 on past behaviors and 16 on current feelings, rated on a 5-point Likert scale from 1 (“completely false”) to 5 (“completely true”). A higher score indicates a higher level of grief.

#### Self-harm and suicidal behaviors

The Ottawa Self-Injury Inventory (OSI, [82]) is a self-report scale that evaluates non-suicidal self-injury (NSSI), which comprises an exploration of the reasons for engaging in NSSI and an assessment of addictive features. The scale comprises four factors: Internal Emotion Regulation, Social Influence, External Emotion Regulation, and Sensation Seeking, and a single Addictive feature factor.

The Columbia Suicide Severity Rating Scale (C-SSRS, [83]) is a clinician-rated questionnaire which can also be used as a self-report. The aim of this scale is to support suicide risk assessment by evaluating suicidal ideation as well as suicidal behavior. Four constructs are measured: Severity of Ideation, Intensity of Ideation, Suicidal Behavior, and Lethality.

#### Motivation to change

The Motivational Ruler is a two-item self-report measure assessing participants’ importance and perceived ability to change. The scale ranges from 0 to 10 with higher scores indicating greater importance or perceived ability, respectively.

#### Satisfaction with treatment

The Client Satisfaction Questionnaire-8 items (CSQ-8, [84]) is a self-report questionnaire assessing satisfaction with health services. Items are answered on a 4-point Likert scale. The CSQ-8 yields a single score measuring overall satisfaction with treatment.

In this trial, we will use a slightly adapted version of the scale. In order to ensure that participants rate the quality of, and satisfaction with the intervention proposed in the context of the trial (SFT or MFT), rather than the whole treatment program, we have replaced “*the services you have received*” by “*the services you have received during your family therapy for anorexia nervosa*” in the initial statement of the scale.

#### Cost calculation

Direct and indirect costs will be recorded with the help of a specific questionnaire, completed in a semi-structured interview. Costs relative to hospitalization, outpatient treatment, other interventions, medication, time spent by the family on treatment, informal care, interruption of schooling, and impact on parents’ professional activities will be taken into account.

### Outcomes

#### Primary outcome

The primary outcome is the evolution of BMI between inclusion and 12 months thereafter.

#### Secondary outcomes

Secondary outcomes are as follows:

- Change from baseline to months 12 and 18 in the overall patient’s clinical outcome (weight outcome, i.e., BMI > 10th percentile, presence of menstruation, GOAS category),
- Change from baseline to months 12 and 18 in the nature and seriousness of patient’s eating disorder symptoms (EDI-2 and EDDS scores),
- Change from baseline to months 12 and 18 in the nature and seriousness of patient’s other psychopathological symptoms (HADS, BDI-13, LSAS-CA-SR, MOCI, BVAQ-B, RSES, OSI, and C-SSRS scores),
- Change from baseline to months 12 and 18 in the nature and seriousness of parents’ psychopathological symptoms (HADS, BDI-13, BVAQ-B, RSES, ECI, EDSIS, and MIV-TIG scores),
- Change from baseline to months 12 and 18 in the quality of family relationships (FAD, SCORE-15, patients’ LEE scale, and parents’ FQ scores),
- Change from baseline to months 12 and 18 in the perceived quality of life (EDQOL and SOFAS scores for patients, WHOQOL-Bref, LHS, and CarerQOL-7D scores for parents),
- Number and duration of hospitalizations after inclusion,
- Patients’ and parents’ satisfaction (CSQ-8 and ECI scores) regarding the intervention,
- Cost of treatment between inclusion, 12 months, and 18 months thereafter.

Other measures will be explored as potential moderators or mediators of treatment outcome.

### Statistics

#### Sample size

One hundred and fifty patients and their parents will be recruited.

Power calculation for the primary endpoint of this study is based on the assumption that MFT is not less effective than SFT if a minimal clinical difference of 0.75 kg/m^2^ in mean change of BMI between baseline and 12 months follow-up cannot be shown between groups. The principal judgement criterion*,* *i.e*., the variation of BMI 12 months after the beginning of treatment, was established after having reviewed family therapy trials and recent trials on AN, whether in the field of family therapy or in other therapeutic trials ([85]). The non-inferiority margin of 0.75 kg/m^2^ is based on clinical experience and is consistent with the scientific literature. The standard deviation of the BMI variation is estimated at 1.45 [85, 86].

Consequently, we determined the size of the sample for a unilateral Student’s *t* test with a significance of 2.5% and a power of 80% with the non-inferiority margin clinically determined for a minimum BMI difference of 0.75 kg/m^2^, assuming the standard deviation to be 1.45.

This procedure led to a required sample size of 60 patients per group. Assuming that the dropout rate is 20% [86], we calculated a sample size of 75 patients per treatment group.

With a total active file of more than 500 patients in the three study sites, we expect to complete the recruitment of 150 eligible patients in 2 years.

#### Statistical methods

Regarding the primary objective, we will compare the evolution of BMI between baseline and after 12 month follow-up in the MFT and SFT groups, using an analysis of covariance (ANCOVA) with BMI at inclusion as a pre-specified co-variable, as well as inpatient status versus outpatient care.

As recommended by the Consolidated Standards of Reporting Trials (CONSORT) guidelines for non-inferiority clinical trials [87], the confidence interval of the intergroup difference of the principal criterion thus obtained will be calculated and represented graphically. The decision to conclude in favor of the non-inferiority of MFT in comparison with SFT will be made only if the upper boundary of the confidence interval is below the chosen non-inferiority threshold of 0.75 kg/m^2^.

It is recommended to carry out a double approach, intention to treat (ITT) and per protocol (PP), for the analysis of the principal criterion in cases of non-inferiority clinical trials [88], as the non-observation of a randomized treatment could reduce differences in efficacy of the two treatments and could therefore bias the trial results. The ITT analysis will include all patients randomized in their randomization group, whatever the treatment they have actually received. The PP analysis will only include patients who have followed at least three sessions without any treatment change or protocol violation. Then, in a sensitivity analysis, we will add age and illness duration to the models as co-variables, as they are important prognostic factors. Finally, if non-inferiority has been demonstrated, the superiority of the MFT treatment over the SFT treatment will be assessed in ITT.

Regarding the secondary outcomes, financial cost and satisfaction will be analyzed for the superiority of the MFT treatment over the SFT treatment. We will compare subject and parent satisfaction with the treatments (as evaluated by the CSQ-8 and ECI) at 12 months using an ANCOVA, with the status of treatment is a co-variable at inclusion (outpatient or inpatient) as a pre-specified co-variable.

In order to compare financial costs between SFT and MFT, we will make an inventory of the treatments, and we will use the average cost for each ambulatory appointment (psychiatrist, psychologist, etc.) and the average treatment cost for hospitalization if necessary during follow-up; the sum will determine the total cost of treatment for each patient. Transport costs and other associated costs will not be estimated, as most patients live near the hospital. Costs for the two treatment groups will be compared using Fisher-Pitman’s test.

An analysis of quantitative variables (assessment scores) will be performed in the same way as for the principal criterion using an ANCOVA, with the value of each respective variable at admission, and for the status of treatment at inclusion (ambulatory care or hospitalization), as co-variables. Morgan- Russell GOAS scores (with the three following outcomes: good, intermediate, and poor) will be compared between SFT and MFT, using Cochran-Armitage’s tendency test. A measure of ordinal association (Somers’ *D*) will be calculated with a confidence interval of 95%. Amenorrhea and other qualitative variables will be compared using Fisher’s exact test. Additionally, for the clinical variables assessing outcome, analyses looking for moderators and treatment effect mediators will be carried out.

#### Data collection and management

Data will be transferred to observation files as it is collected, whether it is clinical or para-clinical data. An electronic data capture system will be used for this trial. Data collected from the subjects will be directly captured into an electronic case report form (e-CRF). The investigator will log onto the clinical study website via a unique access code. Transmitted data will be made secure by a client certificate. Once a patient’s data is entered, the investigator will be able to sign and confirm his/her e-CRF. The system will support and validate data entry. Missing and erroneous data (e.g., out-of-range data) will be automatically detected and reported to the investigator for correction. In addition to this computerized data quality control, a manual quality check will be carried out by a clinical research assistant appointed by the trial Sponsor.

Essential research documents that come within the law on biomedical research will be archived by all parties for 15 years after the end of research.

All video recordings required for conducting supervision sessions will be destroyed at the end of the trial.

This trial is registered with the French National Commission for Data Protection and Liberties (CNIL) under Chapter IX of law no. 78-17 of 6 January 1978, modified by law no. 2016-41 of 26 January 2016, regarding the treatment of personal data for the purpose of scientific research in the health field.

The Sponsor has required an authorization for the processing of collected data, the use of patients’ identifying data (social security number, email address, videotaped sessions conducted with the informed consent of patients and parents), and access to the National Health Insurance Information System (SIIRNAM) database for the cost-efficiency analysis.

## Discussion

International guidelines recommend to involve families in the treatment of AN, but current forms of family therapy are not totally satisfying, with a rate of dropouts and treatment failure that remains relatively high. THERAFAMBEST randomized controlled trial aims to compare multi-family therapy (MFT) in a young outpatient setting to systemic single-family therapy (SFT), as it is presently proposed in the study sites.

The first aim is to examine whether the implementation of MFT is at least as efficacious as that of SFT, in terms of BMI, 12 months after the start of treatment. Secondary aims are to provide new evidence comparing MFT to SFT in terms of efficacy, cost, family satisfaction, and clinical outcome, 12 months after the start of treatment and 6 months after the end of treatment.

The MFT approach has been adapted to AN patients and studies show promising results regarding efficacy, satisfaction with treatment, and prevention of relapse. The current model of MFT, as developed by the Maudsley approach [25], involves an intensive rythm of sessions and strong family motivation to engage in treatment. This modality cannot be easily implemented in our global treatment setting. As a consequence, and given the promising effects of MFT in AN, other French teams have developped their own MFT model in an outpatient setting, and preliminary results are encouraging [38]. However, stronger evidence is still needed, notably a comparison of this new form of family treatment in reference to existing ones.

From our point of view, this study presents some important strengths. The first one is twofold: the design of our study represents the kind of treatments that are provided in “real life,” and these treatments have been manualized. Manualization is not a common practice in real-life care programs, even if it becomes increasingly recommended in order to propose evidence-based and reproducible treatments, where efficacy data can be compared with the international literature. So, for the purpose of this trial, MFT and SFT have been manualized and standardized by experienced psychiatrists and psychologists, with the help of English teams for SFT (Pr. H. Pote [89, 90]) and French teams who developed MFT programs delivered in monthly sessions (i.e. S. Cook-Darzens [91] and Dr. S. Criquillion-Doublet [S. Criquillion-Doublet and E. Martins, personal communication, 2012]) and based on previous manuals developed by different teams ([91]; M. Scholz, M. Rix, K. Hegewald, and K. Grantchev, Treatment manual for multi-family therapy with anorexia nervosa, unpublished manual, 2002, 2003; Maudsley Child and Adolescent Eating Disorders Service, Treatment manual for family therapy — anorexia nervosa, unpublished manual, 2014).

A second strength is that, in addition to standard efficacy outcomes, we plan to conduct a cost-efficiency analysis to compare both therapies in terms of financial costs. Third-party payer perspective, i.e., costs for health care facilities and for national Health care, as well as a societal perspective, i.e., time costs for patients and families and impact on professional activity and quality of life, will be considered. The objective of the study is not only to show that MFT is no less efficacious as SFT, but also to show that it has a lower cost. This would be critical for the evolution and selection of treatments we can propose to patients suffering from AN, given that eating disorders are associated with a substantial economic and social burden and that we can observe a tendency in other countries to limit the length of inpatient stay resulting in more limited weight restoration [92], and a poorer likelihood of successful treatment [93, 94]. This cost-efficiency analysis would help make a rational decision regarding the selection of available effective treatments.

In the same line, the inclusion and exclusion criteria that we have chosen are deliberately broad, to make this trial as naturalistic as possible and to ensure that our sample is representative of the patients we usually follow in our health- care facilities. We expect to define profiles of best responders for each technique. This would enable us to improve therapeutic indications and achieve greater and earlier efficacy, which is predictive of recovery and absence of chronicity.

This trial also presents some challenges we will have to consider. In order to obtain concrete and significant results, we have to recruit 150 patients and their families in a limited timeframe. If projections based on our regular active list of patients in the different study sites make us confident about an effective recruitment, we will consider the option to open a fourth study site in case of difficulties or unexpected delays.

We also wanted to conduct a follow-up evaluation to assess the maintenance of therapeutic results and to have a better evaluation costs in the long term. This will raise the problem of retention. The psychologists and psychiatrists in charge of evaluation are well versed in this problem and have experience in the management of follow-up evaluations of patients and families [86, 95]. Several strategies will be used to maintain the best retention rate possible, such as regular phone calls and communication with the coordinating psychiatrist. A large part of the evaluations will be possible online, with personal access codes, which will enable us to record important information, even if the patient and/or his/her family are not willing to come to the study site for assessment.

A last challenge concerns minimization of therapists’ bias. Indeed, all therapists involved in the trial are trained in both MFT and SFT. We expect to minimize this potential bias by proper training in the use of the therapeutic manuals, regular communication between all therapists, and sessions of supervision.

### Trial status

The trial was registered on ClinicalTrials.gov, NCT03350594, on 22 November 2017; IDRCB number 2016-A00818-43. Recruitment for the trial is ongoing (and started in June 2018). Sessions of MFT and SFT began in September 2018. Recruitment will be completed in September 2021 (approximately).

## Additional file

Additional file 1: SPIRIT 2013 checklist: recommended items to address in a clinical trial protocol and related documents. (DOC 121 kb)

## Abbreviations

AN: Anorexia nervosa
BMI: Body mass index
ED: Eating disorder
EDNOS: Eating Disorder Not Otherwise Specified
MFT: Multi-family therapy
SFT: Systemic single-family therapy
