# Correction to: Multicenter randomized controlled trial on the comparison of multi-family therapy (MFT) and systemic single-family therapy (SFT) in young patients with anorexia nervosa: study protocol of the THERAFAMBEST study

**DOI:** 10.1186/s13063-020-4106-9

**Published:** 2020-02-18

**Authors:** Benjamin Carrot, Jeanne Duclos, Caroline Barry, Leslie Radon, Anne-Solène Maria, Irène Kaganski, Zorica Jeremic, Vesper Barton-Clegg, Maurice Corcos, Malaïka Lasfar, Priscille Gerardin, Aurélie Harf, Marie-Rose Moro, Corinne Blanchet, Stéphane Bahrami, Nathalie Godart

**Affiliations:** 1 Psychiatry Unit, Institut Mutualiste Montsouris, Paris, France; 2 CESP, INSERM, UMR 1018, University Paris-Sud, UVSQ, University Paris-Saclay, Villejuif, France; 3 Univ. Lille, CNRS, CHU Lille, UMR 9193 - SCALab - Cognitive and Affective Sciences, F-59000 Lille, France; 4 Department of Addiction, Eating Disorders Unit, Paul Brousse Hospital, Villejuif, France; 5 Department of Clinical Psychology, Psychopathology, Psychoanalysis - EA 4056 (PCPP), University of Paris Descartes, Paris, France; 6 Department of Medical Pediatrics and Child and Adolescent Psychiatry, Rouen University Hospital and Rouvray Hospital, University of Rouen, Rouen, France; 7 Maison de Solenn, Maison des Adolescents, Hôpital Cochin, Paris, France; 8 Université Paris-Saclay, UVSQ, INSERM, CESP, 78180 Montigny-le-Bretonneux, France; 9 UFR des Sciences de la Santé, Simone Veil (UVSQ), Versailles, France; 10 Fondation de Santé des Etudiants de, France, Paris, France

**Correction to: Trials**

**https://doi.org/10.1186/s13063-019-3347-y**

After publication of our article [1] the authors have notified us of missing to include one name to the authorship list:

“First, S. Bahrami has been involved in the process of our study from the beginning. Second, S. Bahrami worked very hard to achieve the economic part of the protocol - described in the method section ‘Cost-efficiency analysis’. For recall, an economic assessment is being developed in order to take into account the cost of both interventions in our study (MFT and SFT), to identify any possible difficulties in implementing both interventions, to evaluate all treatments and supports that caregivers of patients with eating disorders could for … A cost-efficiency analysis will be conducted on the costs generated by the interventions with a perspective on their impact on patients’ health and families’ quality of life. This methodological choice results from the multi-dimensional consequences of the illness on the patients’ physical and psychiatric health and social integration, and on their families.”

With this addition, the Author’s contribution section is also modified to the below:

BC is the principal investigator of the study. NG is the scientific director of the study and co-investigator. JD is a co-investigator. CBa is the leading biostatistician. SB is in charge of the economic evaluation. NG, BC, JD, and LR designed the study in collaboration with CBl, MC, SB and MRM. JD and LR drafted the original version of the study protocol with the assistance of ASM, NG, VBC and BC. BC, IK, ZJ, and AH are therapists in SFT and MFT. All authors read and approved the final manuscript.
